# The interaction network between intestinal flora and cell death in microecosystem of pan-cancers

**DOI:** 10.7150/ijms.111723

**Published:** 2025-05-16

**Authors:** Yuhan Zhang, Cenzhu Wang, Tingyan Ruan, Shuai Liang, Huning Jiang, Xi Wu, Rui Hou, Hanfang Fan, Huiyu Wang, Junli Ding, Junying Xu

**Affiliations:** The Affiliated Wuxi People's Hospital of Nanjing Medical University, Wuxi People's Hospital, Wuxi Medical Center, Nanjing Medical University

**Keywords:** pan-cancers, intestinal flora, cell death, microecosystem, interaction network.

## Abstract

The human intestinal floras play an important role in human microecosystem, accounting for more than 1,500 species and consisting of beneficial, harmful and neutral bacteria, which take part in regulating the progression of various malignant tumors. Meanwhile, the cell death is a physiological process maintaining biological development and internal environmental homeostasis, including cuproptosis, ferroptosis, disulfidptosis, immunogenic cell death, necroptosis, anoikis, autophagy, pyroptosis and so on. A variety of cell deaths and their related genes have been reported to regulate many malignant tumors. In recent years, there has been increasing interest in understanding the interaction network between intestinal flora and cell death in microecosystem of various malignant tumors. However, this interaction network is still not fully understood and requires further investigation. Therefore, the aim of this study is to explore the potential mechanism network between intestinal flora and cell death in pan-cancers, with the hope that this research could bring a novel insight for the prevention and treatment of tumors.

## Introduction

The intestinal flora and cell death pathways form a complex network that regulates tumor development. Studies show that gut dysbiosis influences tumor progression through multiple mechanisms. Pathogenic bacteria like *Campylobacter jejuni* secrete cytolethal distending toxin (CDT), damaging intestinal epithelial DNA and promoting colorectal cancer^1^. In contrast, probiotics such as *Lactobacillus reuteri* inhibit tumor growth by modulating protein oxidation and ribosome biogenesis^2^.

Meanwhile, programmed cell death pathways play dual roles in the tumor microenvironment. Cuproptosis-related gene expression correlates with breast cancer prognosis^3^. Gasdermin-mediated pyroptosis is abnormally activated in gastrointestinal tumors^4^. Autophagy levels affect tumor cell sensitivity to chemotherapy^5^. Notably, intestinal flora metabolites like butyrate directly regulate cell death processes^6^. Dysbiosis also disrupts tissue homeostasis by influencing anoikis^7^. These interactions may drive cancer progression by modulating inflammatory states, epigenetic modifications, and metabolic reprogramming.

In summary, intestinal flora and cell death pathways critically impact tumor initiation, progression, and treatment. Their interconnected network offers new insights into cancer mechanisms. This review explores their crosstalk to identify novel biomarkers and therapeutic targets. Understanding this regulatory axis may advance pan-cancer treatment strategies targeting microbiota and cell death.

## The interaction network between intestinal flora and ferroptosis in pan-cancers

**Figure 1:**
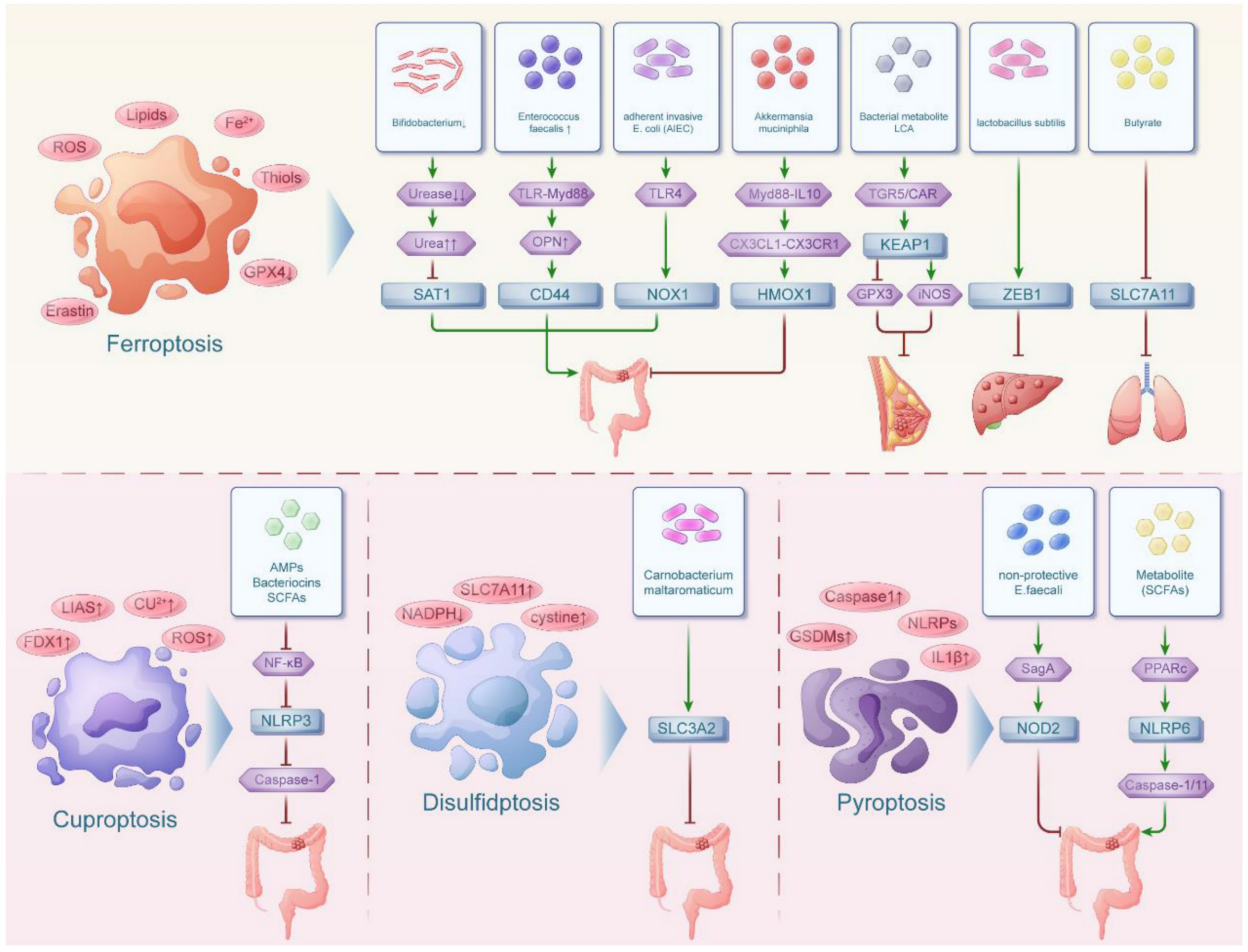
** The interaction network between intestinal flora and ferroptosis, cuproptosis, disulfidptosis, pyroptosis in pan-cancers.** The intestinal floras were associated with ferroptosis in both digestive tumors and non-digestive tumors. Meanwhile, the intestinal floras were related with cuproptosis, disulfidptosis and pyroptosis in mainly digestive tumors.

In 2012, Ferroptosis was coined to describe a form of cell death triggered by iron ions, characterized by uncontrolled lipid peroxidation leading to cell membrane disruption^8^. Over the years, Ferroptosis-related studies have grown exponentially. A multitude of Ferroptosis-related genes have been documented in the literature, including ALOX15, ALOX5, SAT1, SLC7A11, TP53, ZEB1, KEAP1, NOX1, HMOX1, CD44^9^. In recent researches, we could know that SAT1, SLC7A11, TP53, ZEB1, KEAP1, NOX1, HMOX1, CD44 and other Ferroptosis-related genes can interact with the intestinal flora to influence the development of colorectal, lung, breast and pancreatic cancers and other tumors (Figure 1).

### The effect of intestinal flora on ferroptosis in digestive tumors

In the course of colorectal cancer progression, investigations have revealed that a decrease in bifidobacteria, known for their ureolytic capabilities, leads to reduced bacterial urease abundance and elevated urea levels which impairs ureolysis^10^. In the tumor immune microenvironment, urea can enter macrophages, inhibit the binding efficiency of p-STAT1 to the SAT1 promoter region and further bias macrophages toward a pro-tumorigenic phenotype characterized by accumulation of polyamines, which are thought to be immune modulators that inhibit macrophage inflammatory function, leading to colorectal tumorigenesis. Beyond urea-mediated mechanisms, other bacterial products also contribute to tumorigenesis through macrophage modulation. Yuan found that intestinal bacterial products and tumor secretory factors stimulate the myofibroblast-specific MyD88 pathway, provoking osteopontin (OPN) secretion^11^. Elevated OPN levels bind to CD44 and αvβ3 receptors on macrophages, activating the STAT3-PPARγ pathway and promoting M2 macrophage polarization, driving colorectal tumorigenesis. Additionally, studies indicated that *E. faecalis* in the gut induces colitis and upregulates TGF-β expression in intestinal epithelial cells, triggering the transformation of CD44-noncancerous stem cells into CD44+ cancer stem cells, intensifying colorectal cancer development^12^. Recent findings unveiled that adherent invasive *E. coli* (AIEC) binding to intestinal epithelial TLR4 induces DUOX2 and NOX1 expression, heightening epithelial redox activity and ROS production, which accelerates colonic tumorigenesis and progression through ecological dysregulation that impacts microbiota functionality^13^. However, not all microbial-host interactions promote tumorigenesis; some exert protective effects. One study found that Intestinal flora and their products bind to TLR on macrophages and upregulate MyD88 and IL10, thereby upregulating intestinal CX3CL1-mediated upregulation of CX3CR1 receptors on macrophages involved in inducing upregulation of HMOX-1. Increased HMOX-1 and its byproduct carbon monoxide enhance phagocytosis, bacterial elimination, and alter cytokine production to mitigate inflammation and curb colitis-associated cancer formation^14^,^15^.

These microbiota-mediated mechanisms are not limited to colorectal cancer but extend to other digestive tumors. Pancreatic intraepithelial neoplasia (PanIN), the primary precursor lesion to pancreatic ductal adenocarcinoma, is a significant subtype. Chen proposed that intestinal *Lactobacillus* plays a beneficial role in suppressing the expression of epithelial-mesenchymal transition (EMT)-related marker ZEB-1. This leads to a reduction in the quantity and severity of PanIN lesions^16^.

### The effect of intestinal flora on ferroptosis in non-digestive tumors

It was showed that the intestinal microbial metabolite lithocholic acid (LCA) can interact with TGR5 or CAR receptors, influencing the NRF2/KEAP1 system. This interaction results in the downregulation of NRF2 expression and upregulation of KEAP1 expression. The subsequent alterations in NRF2 and KEAP1 expression levels lead to reduced GPX3 expression and enhanced iNOS expression, triggering and regulating the oxidative stress response. This, in turn, exerts a cellular inhibitory effect, slowing down the proliferation of breast cancer cells^17^. While microbial metabolites like LCA show anti-cancer effects in breast cancer, other metabolites demonstrate similar potential in different cancer types through distinct mechanisms. Another study found that during dietary fiber fermentation, the intestinal flora can produce butyrate (a short-chain fatty acid). Butyrate can induce direct binding of ATF3 to the SLC7A11 promoter, thereby modulating the ATF3/SLC7A11 pathway, up-regulating ATF3 expression and decreasing SLC7A11 expression in lung cancer cells and activating erastin-induced iron death in lung cancer cells. Additionally, the combination of butyrate and erastin results in a notable increase in lipid peroxidation and ROS, ultimately reducing cellular viability and promoting cancer cell death for potential anticancer effects^18^.

## The interaction network between intestinal flora and cuproptosis in pan-cancers

Numerous studies highlighted copper's significance as a cofactor for essential metabolic enzymes and its direct involvement in pivotal signaling pathways within tumor cells. When copper metabolism is imbalanced, cells may be severely damaged. For example, excessive copper can cause tumor cells to undergo cell death, which is called cuproptosis. The emergence of cuproptosis as a concept spurred a surge in investigations exploring genes associated with cuproptosis and their impact on tumors. Currently, the known cuproptosis-related genes include NLRP3, ATP7B, SLC31A1, LIAS, LIPT1, DLAT, PDHB, GLS and so on^19^. However, a series of studies elucidated the involvement of NLRP3, a key gene related to cuproptosis, in interactions with intestinal flora, highlighting its role in tumor development (Figure 1).

Damage-associated molecular patterns (DAMPs) and pathogen-associated molecular patterns (PAMPs) can activate NLRP3 inflammatory vesicles. Alternatively, initial signals originating from gut microbial components or endogenous cytokines can induce NF-κB activation, resulting in the upregulation of NLRP3. Augmented NLRP3 levels can recruit caspase-1 to facilitate the formation of NLRP3 inflammasomes, promoting the maturation of pro-IL-1β and pro-IL-18 and driving chronic inflammation in colorectal cancer (CRC)^20^. However, intestinal probiotics residing in the epithelial barrier can downregulate NLRP3-mediated inflammation and stress-induced NF-κB pathways. They stimulate the production of anti-inflammatory cytokines while inhibiting the generation of pro-inflammatory cytokines through the release of antimicrobial peptides (AMPs), bacteriocins, and short-chain fatty acids (SCFAs). These components modulate metabolic processes to mitigate oncogenic inflammation, reducing the risk of carcinogenesis^21^. Beyond general probiotic activity, specific commensal bacteria like *A. muciniphila* demonstrate unique immunomodulatory capabilities in tumor prevention. A study reported that *Akkermansia muciniphila* (*A. muciniphila*) had the capacity to bind to the macrophage pattern recognition receptor TLR2, activating the NF-kB/NLRP3 pathway, promoting the shift towards M1-like tumor-associated macrophages (TAMs), and inhibiting colon tumorigenesis^22^.

## The interaction network between intestinal flora and disulfidptosis in pan-cancers

Disulfidptosis is a newly identified form of programmed cell death. Its hallmark feature involves abnormal disulfide accumulation causing redox imbalance and cytoskeletal protein crosslinking. This discovery offers fresh perspectives for understanding tumorigenesis and developing novel anticancer approaches.

At the molecular level, disulfidptosis involves two critical processes. The first is SLC7A11-dependent cystine uptake. Overactivated SLC7A11 transporter drives excessive cystine influx and reduction to cysteine. This process depletes NADPH and triggers oxidative stress. The second involves disulfide stress. Accumulated cysteine residues form excessive disulfide bonds, disrupting actin and other cytoskeletal proteins. This ultimately leads to cell shrinkage and death 23.

In cancer progression, disulfidptosis exhibits a dual-edged nature. In lung and liver cancers, high SLC7A11 expression promotes tumor growth by suppressing ferroptosis. However, excessive SLC7A11 activation may trigger disulfidptosis, thereby limiting tumor development. Notably, by damaging cytoskeletal structures, disulfidptosis may significantly impair cancer cell migration. This provides potential new strategies against metastasis 24. This unique dual mechanism makes disulfidptosis particularly valuable in cancer therapy research.

Researchers have developed multiple targeted therapies based on disulfidptosis mechanisms. Promising approaches include inducing disulfide stress through glucose metabolism inhibition or direct SLC7A11 targeting. Another strategy combines disulfidptosis inducers with ferroptosis inducers to overcome drug resistance 25. However, clinical translation faces challenges. The overlapping regulatory networks between disulfidptosis and ferroptosis require more precise differentiation methods. Additionally, the lack of specific biomarkers and reliable preclinical models hinders further research progress.

Reviewing the previous literature, the known disulfidptosis-related genes (DRGs) are SLC7A11, SLC3A2, RPN1, NCKAP1, NUBPL, LRPPRC, GYS1, OXSM and so on^26^. A recent study unveiled a significant positive correlation between SLC3A2, a transmembrane glycoprotein, and the abundance of *Carnobacterium maltaromaticum* (*C. maltaromaticum*) within colorectal tumors (Figure 1) 27. Estrogen was found to boost colonic SLC3A2 expression via the bacterial surface protein DD-CPase, facilitating mucosal attachment and colonization by *C. maltaromaticum*. *C. maltaromaticum* significantly increases colonic 25-OHVD3/1,25(OH)2VD3 levels and three enzymes responsible for the biosynthesis of 1,25(OH)2D3 enzymes and significantly activates VDR signaling, which in turn increases the abundance of vitamin D-related metabolites in colonic tissues, resulting in an anti-CRC effect.

Therefore, exploring disulfidptosis-gut microbiota interactions represents a promising new research direction. These studies may lead to breakthroughs in cancer treatment.

## The interaction network between intestinal flora and pyroptosis in pan-cancers

Pyroptosis is a cell death accompanied by an inflammatory response that is triggered by two pathways: activation of the inflammatory cysteine protease Caspase - 1 /4 /5 /11 to cleave GSDMD or activation of the apoptotic cysteine protease caspase-3 to cleave GSDME. Pyroptosis is involved in the development of many diseases. Recent studies have revealed that pyroptosis not only contributes to the development of certain tumors but also exhibits a broader and more rapid tumor-killing effect compared to apoptosis. Consequently, the significance of pyroptosis and its associated genes (such as CASP1, GSDMD, NDO1, NOD2, NLRP3, NLRP6 and so on) in tumor therapy has gained significant attention^28^. In recent years, it was proposed that the intestinal flora may be involved in and influence tumorigenesis and development by mediating the genes related to pyroptosis (Figure 1).

Active enterococci express and secrete orthologs of the NlpC/p60 peptidoglycan hydrolase SagA, which produces immune-active muropeptides. The expression of SagA in non-protective *E. faecalis* has been demonstrated to enhance an anti-PD-L1 response, thereby augmenting cancer immunotherapy. This activity is dependent on the peptidoglycan sensor NOD2. NOD2 serves as a critical pattern recognition receptor for uropeptides and is essential for complementing the antitumor effects of *C. maltaromaticum*-sagA^29^. While NOD2 activation by SagA enhances antitumor immunity, dysregulation of this same receptor can conversely promote tumorigenesis. C. Tikka proposed that NOD2 is a key regulator of the intestinal flora, whereas exposure to elemental arsenic significantly down-regulates the expression of NOD2 proteins and dysfunction or mutation of NOD2 is associated with inflammation. Dysfunction or mutations in NOD2 have been linked to inflammation, leading to dysregulation of the gut microbial ecosystem. This dysregulation can promote tumorigenesis and progression^30^. Beyond NOD2, other NLR family members like NLRP6 also plays pivotal roles in mediating microbiota-induced inflammation and tumorigenesis. NLRP6 belongs to the NLR family and together with NLRP1, NLRP3, NLRP7 and NLRC4 constitutes the ability to construct fully operational inflammatory vesicles. Intestinal microbes and their metabolites have been demonstrated to induce NLRP6 expression by binding to PPARs binding sites, facilitating the assembly of NLRP6 with apoptosis-associated speck-like protein (ASC). This assembly activates Caspase1/11, leading to inflammasome formation. The NLRP6 inflammasome promotes the maturation and release of pro-inflammatory cytokines IL-18 and IL-1β, triggering an inflammatory response that could potentially contribute to colon tumorigenesis^31^.

## The interaction network between intestinal flora and immunogenic cell death in pan-cancers

**Figure 2:**
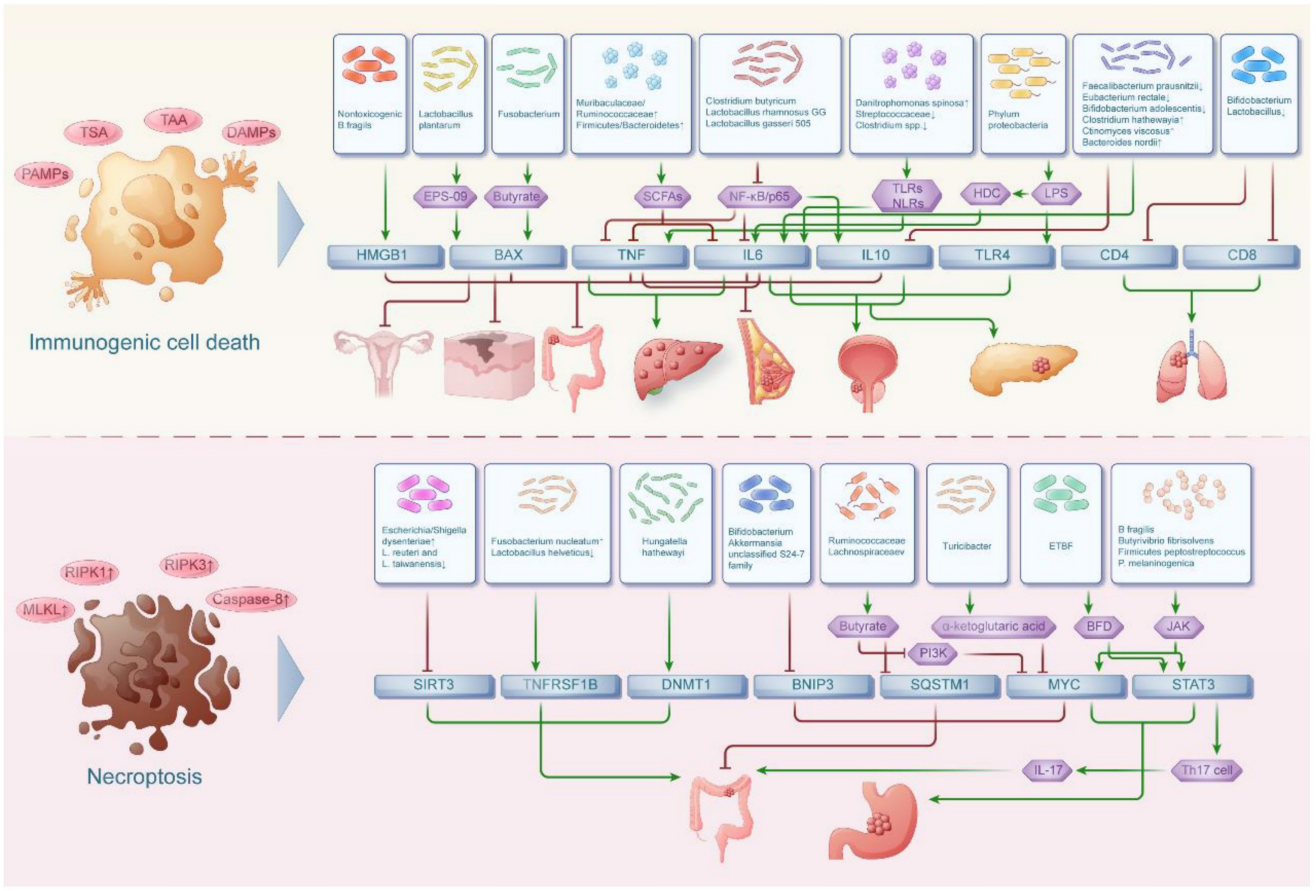
** The interaction network between intestinal flora and immunogenic cell death, necroptosis in pan-cancers.** The intestinal floras were associated with immunogenic cell death in both digestive tumors and non-digestive tumors while the intestinal floras were related with necroptosis in mainly digestive tumors.

Immunogenic cell death (ICD) is a form of cell death characterized by the release of tumor associated antigen (TAA) and tumor specific antigen (TSA), exposing "danger signals" to stimulate the body's immune system to generate an immune response. At the same time, there is a large body of literature revealing the genes involved in immunogenic cell death, encompassing IL6, IL10, TLR4, TNF, HMGB1, BAX, CD4, CD8 and so on^32^. During tumor development, certain intestinal flora can lyse tumor cells to release TAA, express DAMPs and PAMPs and then induce immunogenic cell death in tumor cells. In addition, intestinal flora can also regulate the expression of genes related to immunogenic cell death by influencing the inflammatory response associated with cancer, thus participating in the process of cancer (Figure 2). Among them, the inflammatory effects of the NF-kB signaling pathway are mediated through the induction of downstream cytokines, including COX-2, iNOS and TNFa. It is involved in pro-inflammatory responses to maintain inflammation, promote cell proliferation, angiogenesis and prevent the elimination of cancer cells.

### The effect of intestinal flora on immunogenic cell death in digestive tumors

Recent literature have highlighted the beneficial effects of specific intestinal flora, such as *Clostridium butyricum* (CB)^33^, *Lactobacillus rhamnosus* GG (LGG)^34^,^35^, *Lactobacillus gasseri* 505 (LG)^36^, and *Lactiplantibacillus plantarum*-12 (*L. plantarum*-12)^37^,^38^, in inhibiting colorectal tumorigenesis and development through distinct mechanisms. On the one hand, they can significantly inhibit NF-κ B/p65 signaling pathway activity, thereby down-regulating IL-6, TNF-α and IL-1β and up-regulating IL-10 in the serum of patients with colorectal cancer, which further reduces the inflammatory response associated with colorectal cancer. On the other hand, they can also up-regulate the expression of the pro-apoptotic gene BAX, thereby inducing apoptosis in colorectal epithelial cells and exerting anticancer effects. Related to this, an increase in *Akkermansia muciniphila* following antibiotic treatment was found to elevate mRNA levels of pro-inflammatory cytokines IL-1β, IL-6 and TNF-α, promoting uncontrolled cellular proliferation and furthering colorectal cancer progression^39^. During chemotherapy for colorectal cancer, antitumor adaptive immune responses are known to be activated after chemotherapy with oxaliplatin (OXA). This activation induces ICD in colon cancer cells via the release of DAMPs, such as HMGB1^40^.

While these beneficial bacteria demonstrate protective effects against colorectal cancer, alterations in intestinal flora composition can conversely promote tumor progression in HCC. Patients have a 3-fold increase in the intestinal flora of Danitrophomonas spinosa, a 5-fold decrease in Streptococcaceae, a 5-fold decrease in the abundance of *Clostridium spp.* and an increase in the ratio of *Bacteroides*/*Prevotella*. These alterations have been linked to the activation of inflammatory pathways triggered by Toll-like receptors (TLRs) and NOD-like receptors (NLRs) within the liver. Activation of TLRs and NLRs by gut bacterial products results in heightened levels of pro-inflammatory cytokines IL6 and TNF, fostering an inflammatory environment conducive to cancer development^41^.

Beyond the liver, the influence of intestinal flora extends to other distal organs through intricate inter-organ axes, creating systemic impacts on cancer progression. Researches unveiled the intricate connections between the intestinal-lung axis and the intestinal-pancreatic axis, which are intertwined through lymphatics, the circulatory system, and immune regulation. The intestinal flora can interact to influence the severity of pancreatic cancer and novel coronavirus infection. In pancreatic cancer patients who had been infected by novel coronaviruses, we found that novel coronavirus pneumonia could cause changes in the intestinal flora, such as a decrease in immunomodulatory butyrate-producing flora (*Faecalibacterium prausnitzii*, *Eubacterium rectale*, *Bifidobacterium adolescentis*) and an increase in conditionally pathogenic bacteria (*Clostridium hathewayi*, *Actinomyces viscosus*, *Bacteroides nordii*). These changes were accompanied by heightened expression of pro-inflammatory cytokines IL6 and a decrease in anti-inflammatory cytokine IL10 expression, which may exacerbate the progression of pancreatic cancer^42^.

### The effect of intestinal flora on immunogenic cell death in non-digestive tumors

It was reported that prostate cancer patients on a high fat diet (HFD) reduced gut microbial diversity and ecological dysregulation of the intestinal flora, which results in increased permeability of the intestinal epithelium and leakage of LPS produced, leading to increased systemic levels of LPS. LPS, on the one hand, induces up-regulation of the expression of HDC, a gene responsible for the biosynthesis of histamine, which ultimately leads to a significant increase in histamine production. Binding of histamine to the histamine H1 receptor, which is responsible for local prostate inflammation and immune cell profile growth, can increase the number of myeloid-derived suppressor cells (MDSCs) and activate IL6/STAT3 signaling to promote prostate tumor growth. On the other hand, LPS can also activate TLR4 and induce the LPS/TLR4 signaling pathway to promote prostate cancer development and progression^43^.

Beyond prostate cancer, intestinal flora dysbiosis similarly impacts other malignancies. In the advanced stages of non-small cell lung cancer, the administration of broad-spectrum antibiotics leads to a reduction in beneficial probiotics such as *Bifidobacterium* and *Lactobacillus*, disrupting the balance of the intestinal microbiota. This disruption results in a significant decrease in T cells such as CD4 and CD8, compromising immune cell function. This impairment leads to immune tolerance or escape in non-small cell lung cancer, promoting metastasis of lung adenocarcinoma and lung squamous cell carcinoma^44^.

Conversely, modulating intestinal flora can also exert antitumor effects. Wang discovered that exopolysaccharide (EPS-09) from *Lactobacillus* plantarum WLPL09 exhibit potent antitumor effects in a B16F10 melanoma mouse model. EPS-09 treatment modulated intestinal flora composition in tumor-bearing mice, significantly increasing beneficial bacteria such as Firmicutes, Ruminococcaceae, Lachnospiraceae, and *Ruminococcus*, while reducing potentially pro-inflammatory microbes including *Prevotella*, *Akkermansia*, and Oscillospira. At the molecular level, EPS-09 upregulated the pro-apoptotic gene Bax, activated mitochondrial apoptosis pathways, and induced tumor cell death. These findings suggest EPS-09 may suppress tumor progression through dual mechanisms—the "intestinal flora-immune metabolism" axis and direct gene regulation. It provides new insights for microbial polysaccharide-based anticancer therapies^45^.

The therapeutic potential of microbiota modulation extends to common female malignancies including cervical cancer and breast cancer. In estrogen receptor-negative breast cancer (ER(-) BC), the combined treatment of broccoli sprout (BSp) and ashwagandha (Ash) reshapes gut microbial composition. It increases the abundance of SCFA-producing Muribaculaceae/Ruminococcaceae while maintaining a beneficial Firmicutes/Bacteroidetes ratio. The SCFAs exert antitumor effects by inhibiting HDAC/WNT pathways and reducing pro-inflammatory factors, such as TNF and IL-6. This provides a multi-target therapeutic strategy for the BSp combined with Ash treatment against ER(-) BC^46^. Furthermore, Butyrate, a key short-chain fatty acid produced by gut bacteria like *Lactobacillus* and Fusobacterium, shows strong anti-cervical cancer effects. Using human cervical cancer HeLa and Ca Ski cells, Zhang found butyrate increases apoptosis markers, such as cytochrome C, Caspase 9 and Bax. Concurrently, it specifically inhibits mitochondrial complex Ⅰ activity by reducing NADH and NAD+ levels in mitochondria. Therefore, butyrate triggers apoptosis, effectively blocking cancer cell growth, migration and invasion. These findings reveal butyrate's cancer-fighting mechanism and support its potential as a new cervical cancer treatment derived from gut bacteria metabolites^47^.

## The interaction network between intestinal flora and necroptosis in pan-cancers

In recent years, it was found that necroptosis can be regulated by genetic material like apoptosis, which is a novel mechanism of cell death. It is also known as necroptosis because it is genetically controlled but the cell morphology is necrotic. Recently, the involvement of necroptosis and its related genes in different tumors was investigated. Among them, genes implicated in necroptosis such as BCL-2, TNF, STAT3, SIRT3, TNFRSF1B, MYC, SQSTM1, and BNIP3 have been identified as contributing factors to the development of diverse tumors, modulated by the influence of intestinal flora^48^ (Figure 2).

SIRT3 is a novel colon tumor inhibitor^49^. However, dysregulated gut microbial ecology, characterized by an abundance of pathogenic Escherichia/*Shigella* dysenteriae and a deficiency of beneficial *Lactobacillus reuteri* and *Lactobacillus taiwanensis*, can impair SIRT3 activity^50^. Moreover, TNFRSF1B is one of the receptors for tumor necrosis factor (TNF). It not only supports cell survival and proliferation but also shows a positive correlation with metastasis in tumors. Currently, a negative relationship exists between TNFRSF1B gene expression and the abundance of *Lactobacillus helveticus*. Therefore, introducing *Lactobacillus helveticus* or a probiotic blend containing it can downregulate TNFRSF1B, thus reducing colorectal cancer risk^51^. Other related literatures indicated that diets rich in fat and sugar may lead to an excess of inflammatory bacteria, including *Fusobacterium nucleatum*, which causes increased levels of intestinal inflammatory TNFRSF1B, thus creating a setting of intestinal inflammation and hastening the advancement of tumors^52^. In addition, entero toxigenic *bacteroides fragilis* (ETBF) were found to produce the major virulence factor, *B. fragilis* toxins (BFT), which induces cleavage of E-cadherin, activates the Wnt/β-catenin signaling pathway, induces transcription and translation of c-Myc and promotes the proliferation of colonic epithelial cells (CECs). BFT additionally activates the STAT3 regulatory Th17 cells, which produce IL-17 and promote tumor development^53^. In contrast to these pro-tumorigenic mechanisms, certain bacterial strains demonstrate remarkable anti-tumor potential through metabolic regulation. The presence of *Turicibacter* has potential implications for the prevention and treatment of intestinal tumors. Recently, it was reported in the literature that the combination of *Turicibacter* and the medicinal fungus *Antrodia camphorata* could emerge as a viable option for supplementary and alternative cancer treatments. *Turicibacter* may result in a marked rise in α-ketoglutaric acid levels. α-ketoglutaric acid can relieve intestinal inflammation and increase the abundance of anti-inflammatory and SCFA-producing bacteria. Higher levels of α-ketoglutarate effectively inhibited the expression of Wnt signaling-related proteins and downstream genes such as c-Myc, which suppressed the Wnt/β-catenin signaling pathway. This inhibited the proliferation, metastasis and epithelial-mesenchymal transition of CRC cells and induced apoptosis^54^.

Similar pro-inflammatory pathways are also activated in upper digestive tumors through altered bile acid metabolism. Wang concluded that bile acids were significantly increased in the gastric juices of patients with bile reflux gastritis and gastric cancer. The increase in BAs was associated with an increase in the abundance of LPS-producing bacteria, such as *Pseudomonas melaninogenica* (PM). Taurodeoxycholic acid (TDCA) was significantly positively correlated with LPS-producing bacteria in the gastric juices of these patients. LPS produced by PM promote the phosphorylation of JAK, which enhances STAT3 expression. STAT3 activation is a common pro-inflammatory oncogenic feature in many solid tumors. In addition, mRNAs of STAT3 target genes such as c-Myc were upregulated accordingly. These results suggest that TDCA and LPS may promote gastritis by activating the IL-6/JAK1/STAT3 signaling pathway, which promotes the proliferation of gastric epithelial cells and further develops to form gastric carcinoma^55^. In addition to this, it was found that alterations in intestinal bacterial profiles (*B. fragilis*, *Butyrivibrio fibrisolvens* and Firmicutes *Streptococcus digestans* increase in relative abundance) in the deficiency of vitamin D receptor also hyperfunction JAK and enhance STAT3 expression in the organ, leading to hyperfunction of JAK/STAT3 signaling, which promotes tumor development^56^.

In addition, dietary fiber can be fermented by gut microorganisms to produce SCFAs such as butyrate. Butyrate can inhibit NK cell by inhibiting mTORC1 and c-MYC-dependent signaling. The mTORC1-c-MYC axis controls the expression of the metabolic enzyme methionine adenosyltransferase 2α (MAT2A). MAT2A activity is required to support protein synthesis and tumor growth. Therefore, butyrate inhibits the expression of this enzyme, thereby inhibiting tumor growth. In addition to this, butyrate-treated NK cells were able to differentially express the ubiquitin-binding selective autophagy receptor SQSTM1, thus exerting NK cell immunomodulatory activity^57^. Another study found that a fiber-rich diet increases the relative abundance of *Bifidobacterium*, *Akkermansia* and unclassified S24-7 family, significantly downregulates the autophagy markers BNIP3, thereby downregulating the autophagy pathway to inhibit proteolysis. This dietary modulation alters the composition of the intestinal flora, potentially attenuating skeletal muscle loss in colorectal cancer cachexia^58^.

More so, it was showed that butyrate could inhibit miR-92a transcription, increase PTEN expression and antagonize the effect of PI3K by inhibiting the expression of c-Myc. This can reduce colon cancer cell proliferation and stimulate apoptosis^53^,^59^. The above studies suggest that intestinal flora can mediate the involvement of necroptosis-related genes including STAT3, MYC and SQSTM1 in colorectal tumor development through the Wnt/β-catenin signaling pathway, IL-6/JAK1/STAT3 signaling pathway and mTORC1-c-MYC axis respectively.

There are even studies suggesting that changes in the microbiome composition and aberrant hypermethylation of tumor suppressor genes promoters are two important markers of colorectal cancer. *Hungatella hathewayi* (*H. hathewayi*) was identified as a methylation-regulating bacteria. And DNMT1 is responsible for the DNA methylation activity required to maintain replicative methylation patterns during DNA replication. *H. hathewayi* can increase the expression of DNMT1 to regulate host methylation, which can promote intestinal cell proliferation and drive colorectal cancer development^60^.

## The interaction network between intestinal flora and anoikis in pan-cancers

**Figure 3:**
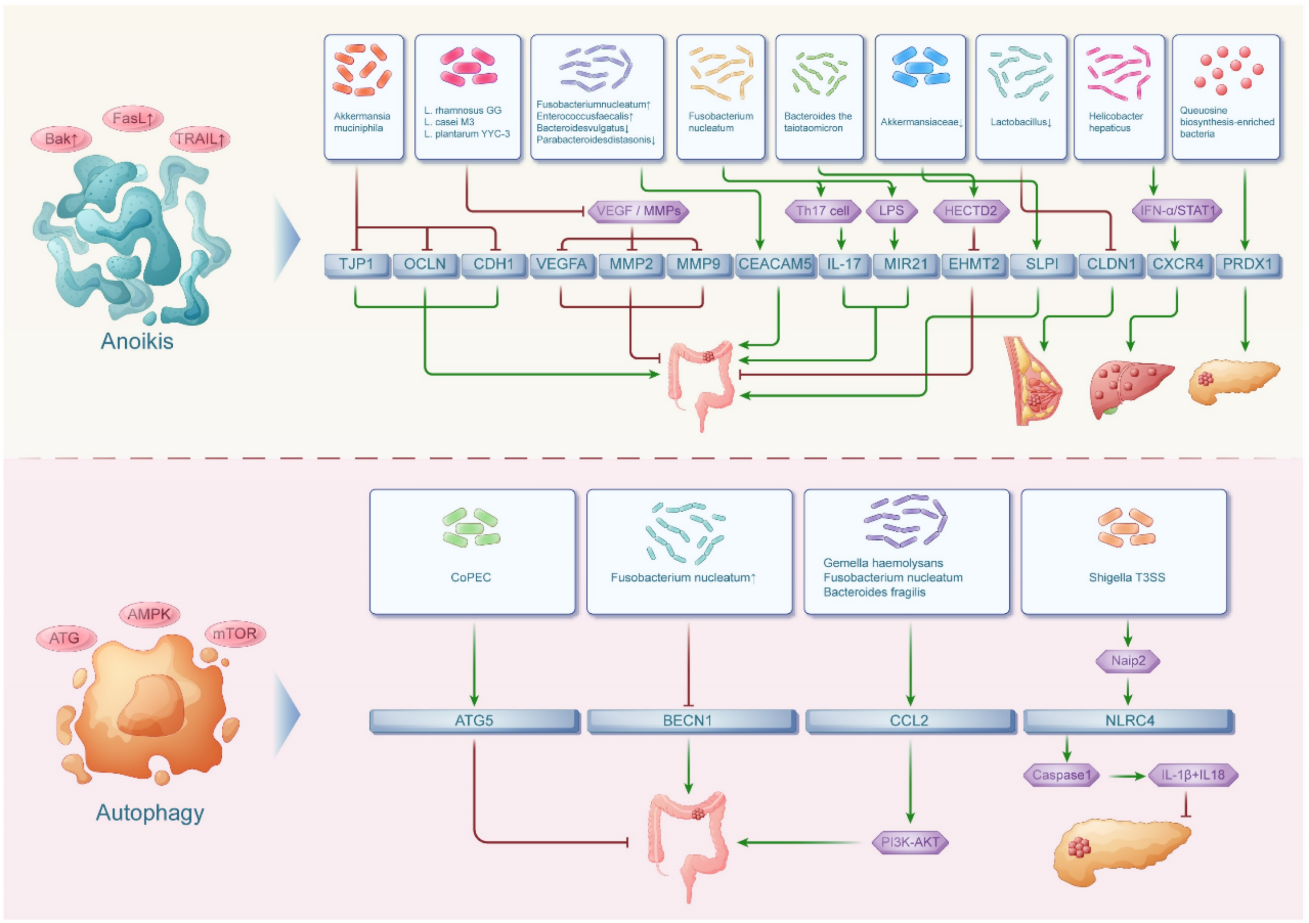
** The interaction network between intestinal flora and anoikis, autophagy in pan-cancers.** The intestinal floras were associated with anoikis in both digestive tumors and non-digestive tumors while the intestinal floras were related with autophagy in mainly digestive tumors.

Anoikis is a form of cell death that occurs when cells lose attachment to the extracellular matrix, which disrupts intercellular and cell-matrix signaling pathways. At the same time, intestinal bacteria were implicated in impacting anoikis and its associated genes (TJP1, OCLN, CDH1, VEGFA, MMP2, MMP9, PTEN, CEACAM5, IL17, MIR21, EHMT2, SLPI, CLDN1, CXCR4, PRDX1)^58^ which collectively play a crucial role in malignant tumor metastasis and cell survival (Figure 3).

### The effect of intestinal flora on anoikis in digestive tumors

*Akkermansia muciniphila*-mediated rebuilding of the intestinal microbiota after antibiotics disrupts the intestinal barrier, thereby exacerbating CRC. Meanwhile the expression of TJP1 (gene encoding tight junction protein 1), OCLN (gene encoding occludin) and CDH1 (gene encoding E-calcium mucin) were all decreased, suggesting that the increase in *Akkermansia muciniphila* after antibiotic treatment induced further damage to the intestinal barrier, thereby exacerbating CRC^39^. Apart from that, metabolite secretions of L. rhamnosus GG, L. casei M3 and *L. plantarum* YYC-3 have growth inhibitory and anti-metastatic activities on cells of colon cancer cell lines. VEGF is known to be a major pro-angiogenic factor, inducing mitosis and regulating endothelial cell permeability. In addition, MMPs are widely used to indicate the metastatic ability of colon cancer cells. Among them, MMP2 and MMP9 play important roles in promoting tumor cell metastasis. *L. plantarum* YYC-3 has demonstrated remarkable efficacy in suppressing CRC cell growth, invasion and migration, significantly reducing the expression of key genes like MMP2, MMP9 and VEGFA that are crucial for tumor angiogenesis and metastasis^61^,^62^.

Beyond direct bacterial effects, microbial receptors also play pivotal roles in shaping the tumor-promoting microbiome. Increased abundance of the microbial receptor CEACAM5 alters the intestinal microbiome by favoring bacterial species linked to colon tumor development, such as *Fusobacterium nucleatum* and *Enterococcus faecalis* while reducing beneficial bacterial numbers like *Bacteroides vulgatus* and *Parabacteroides distasonis*, thus promoting tumorigenesis^63^. In addition, it was found that *F. nucleatum* increased expression of colonic Th17 cells and their intracellular pro-inflammatory cytokines IL-17A and IL-17F, thereby enhancing intestinal tumorigenesis. Moreover, LPS produced by *F. nucleatum* activate TLR signaling, leading to NF-κB activation and MIR21 expression. These can potentially influence the development of colitis-associated cancers^64^.

In contrast to these pro-inflammatory mechanisms, certain microbial metabolites exhibit anti-tumor properties through epigenetic regulation. The intestinal flora metabolite propionate upregulates HECTD2 and promotes proteasomal degradation of EHMT2 through post-translational modifications. Down-regulation of EHMT2 up-regulates TNFAIP1 expression. TNFAIP1 is a direct target of EHMT2 to induce apoptosis in colorectal cancer cells. Therefore, microbiome-derived propionate plays a pivotal role in regulating colon cancer progression through the HECTD2-EHMT2-TNFAIP1 pathway^65^.

Moreover, some host defense proteins can be hijacked by tumors to create an immunosuppressive microenvironment. SLPI is a highly conserved pleiotropic protein that modulates the inflammatory response and protects the host from infection. These properties enable tumor cells to use SLPI to establish an immune surveillance microenvironment. And the upregulation of SLPI can accelerate tumor development, promote tumor metastasis and induce angiogenesis. Accordingly, Liu found that excess dietary iron significantly depleted *Akkermansia*ceae. Reduced intestinal *Akkermansia*ceae levels exacerbated intestinal barrier dysfunction, stimulated intestinal epithelial permeability and led to leakage of luminal bacteria. This prompts epithelial cells to release more SLPI to combat leaking bacteria and limit inflammation. Upregulated SLPI acts as a pro-tumorigenic factor to promote colorectal tumorigenesis^66^.

The gut-liver axis represents another critical interface where microbiota influence cancer development. Han found that *Helicobacter hepaticus* can colonize the lower colon of HBV-infected HCC mice during HCC development and does not leave the intestine. *Helicobacter hepaticus* might act synergistically with HBV to recruit NK cells that secrete IFN-γ. IFN-γ induces an increase in CXCR4 expression through the IFN-γ/p-STAT1 axis and generates a detrimental immune microenvironment, thus exacerbating the tumorigenesis of HBV-associated hepatitis^67^.

Dietary factors can further modulate these microbiota-cancer interactions through metabolic reprogramming. PRDX1 is known to be an antioxidant protein that protects tumor cells from chemotherapy-induced oxidative stress. However, high-fat diets can lead to the accumulation of the intestinal microbial metabolite Queuosine, which elevates the expression of PRDX1. The upregulation of PRDX1 protects tumors from chemotherapy-induced oxidative stress and leads to treatment resistance in pancreatic cancer^68^.

### The effect of intestinal flora on anoikis in non-digestive tumors

Patients with breast tumors have a lower relative abundance of *Lactobacillus* in the gut. Reduced intestinal *Lactobacillus* can decrease the expression of the tight junction proteins CLDN1, which compromises intestinal barrier function. This contributes to increased exposure of the intestinal epithelium to bacteria and elevating the risk of bacterial translocation^69^.

## The interaction network between intestinal flora and autophagy in pan-cancers

Autophagy is a membrane-mediated process when substances in the cytoplasm are transported to lysosomes for degradation. Autophagy is widely present in both anabolic and catabolic processes in the human body. Meanwhile, autophagy-related genes, including ATG5, BECN1, CCL2, PINK1, NLRC4, HGF and MET, play a significant role in regulating inflammatory responses, intestinal bacterial infections and tumor development^70^ (Figure 3).

Patients with Colibactin-producing *Escherichia coli* (CoPEC) colonization in the colon exhibit higher mRNA levels encoding proteins associated with autophagy in the colonic mucosa. Scholars such as C. Lucas found that knockdown of ATG5 in cells can result in elevated secretion of pro-inflammatory cytokines IL6, IL8 and TNF, along with DNA double-strand breaks. Conversely, it can also decrease IL10 mRNA levels and markers of DNA repair. Thus it is clear that CoPEC infection can activate specific autophagy in intestinal epithelial cells, thereby eliminating intracellular bacteria, inhibiting inflammation, repairing DNA damage and preventing colorectal carcinogenesis^71^.

While CoPEC-induced autophagy shows protective effects, other bacteria like *F. nucleatum* demonstrate contrasting roles in colorectal carcinogenesis. *F. nucleatum* emerges as a potential driver of colorectal carcinogenesis. However, related studies uncovered a negative correlation between BECN1 expression, a key initiator of autophagy body formation, and the abundance of *F. nucleatum* in colorectal cancer tissues. This suggests that autophagy induced by BECN1 may play a crucial role in removing invasive microorganisms such as *F. nucleatum* from the tumor microenvironment, thereby reducing the risk of colorectal carcinogenesis^72^.

Among cytokines, C-C Motif Chemokine Ligand 2 (CCL2) is produced by tumor cells and a variety of other cells in the host microenvironment, including adipocytes. CCL2, produced by tumor cells and various host microenvironment components like adipocytes, can directly stimulate cancer cells and bolster proliferation through the PI3K-AKT phosphorylation pathway^73^. Recently, the correlation between circulating levels of CCL2 and the presence of *Gemella haemolysans*, *F. nucleatum* and *Bacteroides fragilis* was investigated in the literature. Ultimately, it was found that the increase in CCL2 levels was influenced by the increase in bacterial abundance. And increased CCL2 expression can promotes CRC progression and malignancy^74^,^75^.

In pancreatic cancer, bacterial components can also modulate cell death through inflammasome activation. In addition, expression of *Shigella* T3SS rod protein MxiI induces activation of NLRC4 inflammasome. Subsequently, NLRC4 inflammasome bodies activate caspase-1. The activated caspase-1 can lead to the release of mature IL-1β and IL-18 and induce apoptosis in pancreatic cancer cells^76^,^77^.

## Conclusion

Intestinal flora is both an important pathogenic factor in various tumor diseases and a potential target for cancer therapy. Various cell death also play a non-negligible role in cancer development. This paper summarizes and depicts the strong correlations and related networks between intestinal flora and cell death. Meanwhile, the mechanism by which intestinal flora affects tumorigenesis, progression and prognosis by inducing different types of cell death and their related genes is also discussed in detail.

The discovery of the intestinal flora-cell death network offers fresh insights into cancer research. This complex regulatory system suggests that the co-evolution of microbiomes and host cell death pathways may follow universal patterns in tumorigenesis. From a translational medicine perspective, deciphering this network could reveal new cancer biomarkers—and more importantly, universal therapeutic targets across cancer types. For example, small-molecule drugs could be developed to target microbial metabolites that regulate cell death. Dietary interventions may also enhance tumor cell sensitivity to programmed death. Additionally, this field may advance precision medicine by exploring "microbiome-host interactions," providing new strategies for personalized cancer treatment. Future research should integrate multi-omics approaches to advance exploration across three key dimensions: molecular mechanisms, animal models, and clinical translation—ultimately bridging the gap between fundamental discoveries and real-world medical applications.

Despite its therapeutic potential, clinical translation faces major hurdles. Biologically, the system is highly complex: different strains of the same bacterial species can have opposing effects on cell death. For instance, *E. coli* K12 promotes ferroptosis, while its pathogenic strain O157:H4 inhibits it. Tumor microenvironments also show spatial heterogeneity in microbial distribution, leading to varying cell death response. Technologically, current sequencing methods miss low-abundance functional bacteria, and organoid models replicate less than 5% of gut microenvironment components^78^. Clinically, treatment efficacy varies widely due to individual differences. To overcome these challenges, researchers are developing innovative tools like 18F-labeled SCFA PET tracers and standardized microbiome libraries covering 90% of population-specific strains^79^. These advances may provide critical support for future breakthroughs.

## Abbreviations

CDT: Cytolethal distending toxin
GSDM: Gasdermin
TGF-β: Transforming growth factor β
CSCs: CD44-noncancerous stem cells
AIEC: Adherent invasive *E. coli*
PanIN: Pancreatic intraepithelial neoplasia
EMT: Epithelial-mesenchymal transition
LCA: Lithocholic acid
DAMPs: Damage-associated molecular patterns
PAMPs: Pathogen-associated molecular patterns
CRC: Colorectal cancer
AMPs: Antimicrobial peptides
SCFAs: Short chain fatty acid
*A. muciniphila*: *Akkermansia muciniphila*
TAMs: Tumor-associated macrophages
SLC7A11: Solute carrier family 7 member 11
DRGs: Disulfidptosis-related genes
*C. maltaromaticum*: *Carnobacterium maltaromaticum*
PPARs: Peroxisome proliferators-activated receptors
ASC: Apoptosis-associated speck-like protein
ICD: Immunogenic cell death
TAA: Tumor associated antigen
TSA: Tumor specific antigen
CB: *Clostridium butyricum*
LGG: *Lactobacillus rhamnosus* GG
LG: *Lactobacillus gasseri* 505
*L. plantarum*-12: *Lactiplantibacillus plantarum*-12
OXA: Oxaliplatin
HCC: Hepatocellular carcinoma
TLRs: Toll-like receptors
NLRs: NOD-like receptors
HFD: A high fat diet
MDSCs: Myeloid-derived suppressor cells
EPS-09: Exopolysaccharide
BC: Breast cancer
BSp: Broccoli sprout
Ash: Ashwagandha
Caspase: Cysteine-containing aspartic protein hydrolase
TNF: Tumor necrosis factor
ETBF: Entero toxigenic *bacteroides fragilis*
BFT: *B. fragilis* toxins
CECs: Colonic epithelial cells
TDCA: Taurodeoxycholic acid
PM: *Pseudomonas melaninogenica*
MAT2A: Methionine adenosyltransferase 2α
*H. Hathewayi*: *Hungatella hathewayi*
*F. nucleatum*: *Fusobacterium nucleatum*
FR: Fiber-rich
LBP: Lipopolysaccharide-binding protein
CoPEC: Colibactin-producing *Escherichia coli*
CCL2: C-C Motif Chemokine Ligand 2
